# Study on the Gas-Chromic Character of Pd/TiO_2_ for Fast Room-Temperature CO Detection

**DOI:** 10.3390/molecules29163843

**Published:** 2024-08-13

**Authors:** Xinbao Li, Kai Sun, Ying Chen, Ye Yuan

**Affiliations:** 1 College of Energy Environment and Safety Engineering, China Jiliang University, Hangzhou 310018, China; 2 Faculty of Maritime and Transportation, Ningbo University, Ningbo 315211, China

**Keywords:** noble metal, TiO_2_, carbon monoxide, gasochromic, DFT

## Abstract

As a widely used support, TiO_2_ has often been combined with Pd to form highly sensitive gas-chromic materials. Herein, we prepared a series of Pd/TiO_2_ catalysts with different Pd content (from 0.1 to 5 wt.%) by the impregnation method for their utilization in fast room-temperature CO detection. The detection was simply based on visible color change when the Pd/TiO_2_ was exposed to CO. The sample with 1 wt.% Pd/TiO_2_ presented an excellent CO gasochromic character, associated with a maximum chromatic aberration value of 90 before and after CO exposure. Systematic catalyst characterizations of XPS, FT-IR, CO-TPD, and N_2_ adsorption–desorption and density functional theory calculations for the CO adsorption and charge transfer over the Pd and PdO surfaces were further carried out. It was found that the interaction between CO and the Pd surface was strong, associated with a large adsorption energy of −1.99 eV and charge transfer of 0.196 *e*. The color change was caused by a reduction in Pd^2+^ to metallic Pd^0^ over the Pd/TiO_2_ surface after CO exposure.

## 1. Introduction

Carbon monoxide (CO) is a poisonous gas often produced in industrial production and daily life. CO is the product of the incomplete combustion of carbon-containing substances [1]. People find it difficult to detect by their sense of sight and smell because of its colorless and odorless characteristics. It is easy to have a huge negative impact on health and life when humans are exposed to CO for a long time. Thus, detecting CO leakage over time in industrial production and in everyday life is necessary [2,3,4,5,6,7].

Recently, transition metal oxide has been widely used in a variety of gas-sensing applications [8]. Transition metal oxide can interact with redox gases due to their one or more intermediate valence states. This characteristic results in reversible color change [9,10,11,12]. Sambare et al. investigated the adsorption characteristics of CO on transition metals (Co, Cr, Cu, Mn, Mo, Nb) doped with BiFeO_3_ perovskites via density functional theory (DFT) calculations and found that Mo-doped BiFeO_3_ exhibited high CO adsorption capabilities, demonstrating its good potential in CO detecting [13]. Promthong and colleagues investigated the adsorption of CO and CO_2_ on transition-metal-doped graphene nanoflakes using density functional theory and found that chromium-doped graphene nanoflakes exhibited the highest adsorption capabilities [14]. TiO_2_ has a large specific surface area and a high dielectric constant. Therefore, TiO_2_ has better electrochromic properties and sensing properties compared with other transition metal oxides such as WO_3_ and MoO_3_ [15]. Most CO sensors based on transition metal oxides need to be heated before they can operate [16], so suitable catalytic materials are required to promote the color reaction between CO and transition metal oxides. Noble metals, such as Au, Ag, Pd, and Pt, have excellent catalytic properties. They are common materials for various gas sensing [17,18,19,20,21,22,23]. Pd can undergo redox reactions with CO at low temperatures to generate carbon dioxide [24,25]. To date, supporting Pd particles on reversible discoloration carriers can improve the sensitivity and reaction efficiency of CO sensors [26,27,28]. Thus, Pd is often used in conjunction with MOS sensors to detect CO at lower temperatures [29]. However, the price of Pd as a noble metal has been high. It is necessary to explore a material that can still maintain high gas-chromic performance for CO detection at relatively low Pd content levels.

Here, we reported a gas-chromic material based on Pd/TiO_2_ for the detection of CO at room temperature. The color reaction of Pd/TiO_2_ with different Pd contents in the CO environment was studied systematically. In addition, three other noble metal materials were prepared for comparison with Pd. The adsorption energy of CO on these noble metal surfaces was analyzed by DFT. The experimental results showed that Pd had better adsorption energy and Pd/TiO_2_ showed stronger gas-chromic properties. This result makes this material a promising gas-chromic material.

## 2. Results and Discussion

### 2.1. Characterization of Pd/TiO_2_

**Figure 1:**
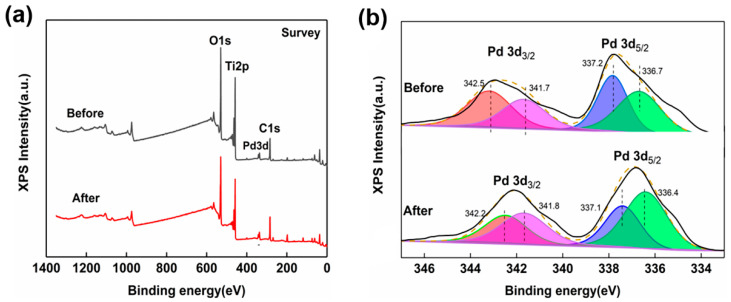
(**a**) Surface chemical state XPS determination of 1 wt.% Pd/TiO_2_, and (**b**) its Pd 3d peaks pre- and post-exposure to CO. The green and pink peaks represent Pd^0^, and the purple and orange peaks represent Pd^2+^.

Figure 1a presents the typical XPS spectra of 1 wt.% Pd/TiO_2_ pre- and post-CO exposure, indicating the changes in elemental composition and chemical state. As can be seen from the figure, Pd/TiO_2_ contains Ti and O elements before and after exposure to CO and has obvious characteristic peaks of C and Pd. The presence of the C element is due to the adsorption of CO by Pd/TiO_2_ during CO exposure. The presence of the Pd element proves the successful loading of Pd on TiO_2_. The XPS spectra of Pd 3d for the catalysts are shown in Figure 1b. In Figure 1b, the 3d orbital of Pd has two independent orbitals of Pd 3d_5/2_ and Pd 3d_3/2_, and the binding energy difference between these two orbitals is about 5 eV [30,31]. The characteristic peaks of the binding energy of ~336 eV and ~337 eV correspond to Pd^0^ and Pd^2+^, respectively. After CO exposure, the characteristic peak shifted to the right by 0.9 eV, indicating a reduction in Pd^2+^ to metallic Pd. The relative peak area ratio of Pd^0^/Pd^2+^ in the after-CO exposure sample was significantly larger than that in the before-CO exposure sample (Figure 1b). In the XPS studies previously carried out by Zedan et al. for Cu-doped Pd/TiO_2_, the binding energies of metallic Pd were identified to be 337.1 eV (Pd 3d_5/2_) and 342.5 eV (Pd 3d_3/2_), which were in good agreement with our results [32].

**Figure 2:**
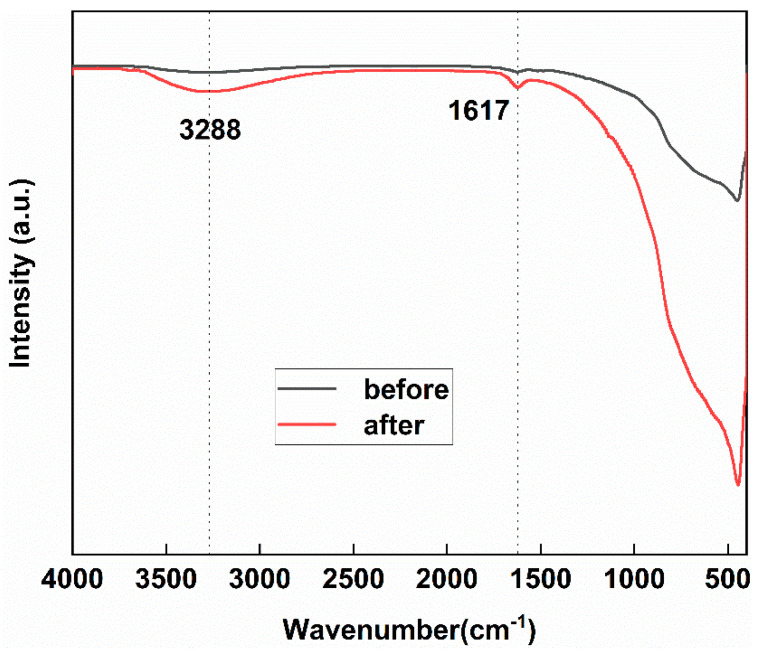
FT-IR spectra of 1 wt.% Pd/TiO_2_ of pre- and post-exposure to CO.

Figure 2 shows the FT-IR spectra before and after the reaction of 1 wt.% Pd/TiO_2_ with CO. There are two distinct bands between 1000 cm^−1^ and 4000 cm^−1^. The infrared band at 3288 cm^−1^ belongs to the O-H group vibration on the catalyst surface. After calcination, part of the H remains in the material and enters the TiO_2_ crystal lattice to form Ti-OH. The infrared band at 1617 m^−1^ is caused by C=O stretching. There are more O-H groups at 3288 cm^−1^ after the reaction of 1 wt.% Pd/TiO_2_ with CO, indicating that there is a strong cooperative interaction between CO and Ti-OH. More C=O groups appeared after the reaction at 1617 cm^−1^, indicating that CO is adsorbed and oxidized in large quantities on the catalyst surface [33].

**Figure 3:**
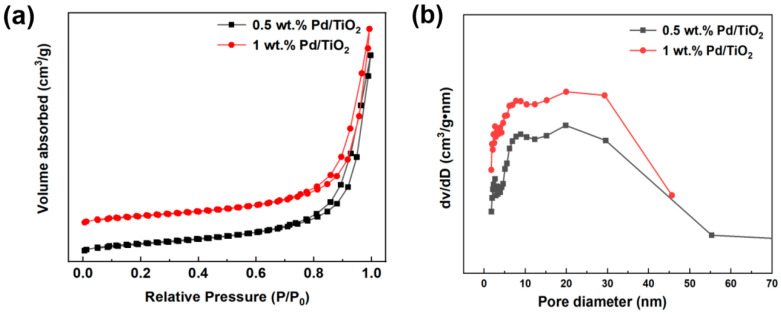
(**a**) N_2_ adsorption–desorption isotherms of the catalysts of 0.5 wt.% and 1 wt.% Pd/TiO_2_, and (**b**) their corresponding pore size distributions.

The N_2_ adsorption–desorption analysis of Pd/TiO_2_ with different Pd loading amounts was carried out, and the N_2_ adsorption–desorption isothermal curves are shown in Figure 3a. A hysteresis loop occurred at a relative pressure of about 0.7, and capillary condensation occurred. Both 0.5 wt.% Pd/TiO_2_ and 1 wt.% Pd/TiO_2_ showed a type IV adsorption isotherm and a type H_3_ hysteresis loop [34]. The corresponding pore size distribution of the catalysts is shown in Figure 3b. The pore sizes of 0.5 wt.% Pd/TiO_2_ and 1 wt.% Pd/TiO_2_ are mainly concentrated in the mesoporous region, and the pore sizes are distributed at 20–25 nm.

Table 1 exhibits the textural properties of 0.5 wt.% Pd/TiO_2_ and 1 wt.% Pd/TiO_2_. The specific surface area and pore volume of catalysts increased with an increase in Pd loading. The specific surface area increased from 75.2 m^2^·g^−1^ to 76.8 m^2^·g^−1^, and the pore volume increased from 0.44 cm^3^·g^−1^ to 0.45 cm^3^·g^−1^. The pore size distribution and pore data comprehensively verified that many mesopore pores were introduced by increasing Pd loading. The pore size distribution map and pore data comprehensively verified that a large number of mesopores were introduced by increasing the Pd loading. This result was beneficial to improve the adsorption and oxidation of CO by Pd/TiO_2_ [35].

**Figure 4:**
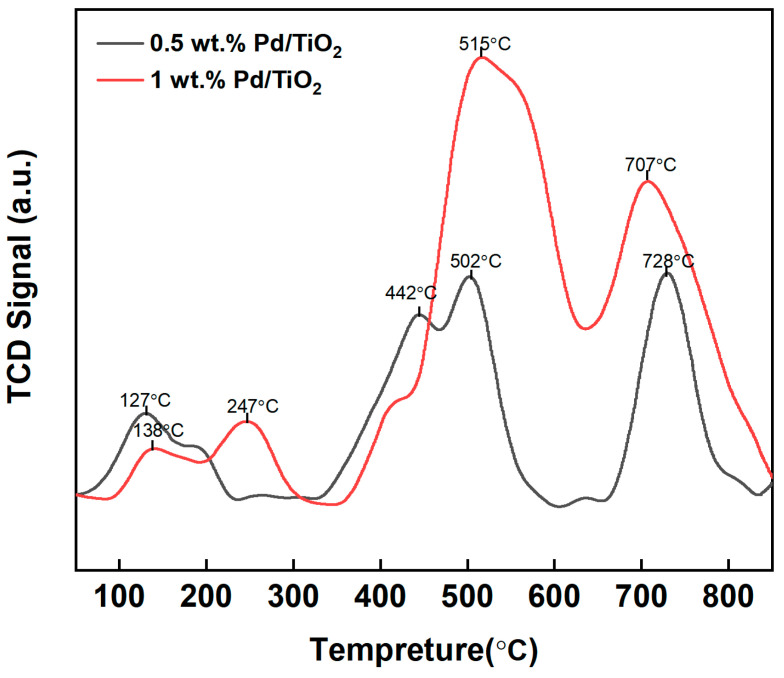
CO-TPD profiles of 0.5 wt.% and 1 wt. Pd/TiO_2_ for the indication of CO-catalyst interaction.

The interactions between CO and the 0.5 wt.% and 1 wt.% Pd/TiO_2_ catalysts were detected by CO-TPD, and the corresponding CO desorption profiles are shown in Figure 4. The desorption peaks of both catalysts mainly ranged at 340–610 °C and at 650–820 °C, which corresponded to moderate and strong CO surface interactions [36]. It was obviously found that the amount of CO desorption on the surface of 0.5 wt.% Pd/TiO_2_ was much lower than that on the 1 wt.% Pd/TiO_2_. As a result, the interaction between CO and the Pd/TiO_2_ was enhanced by increasing the Pd content, which provided more active sites for CO adsorption and, thereafter, improved the redox properties of Pd/TiO_2_. Therefore, the CO gasochromic performance can be enhanced. Compared with the sample of 0.5 wt.% Pd/TiO_2_, the number of obvious CO desorption peaks was reduced from three to two in the sample of 1 wt.% Pd/TiO_2_, while its moderate-temperature CO desorption peak was shifted to the right from 502 °C in the 0.5 wt.% Pd/TiO_2_ to 515 °C. This indicated that the interaction between CO and the catalyst at a moderate temperature range was enhanced. Liu et al. previously carried out DFT calculations of CO adsorption over Pd/graphene and found that CO stretching was redshifted during CO oxidation, showing the positively charged nature of the Pd atoms [37]. Their DFT calculations were consistent with our CO-TPD measurement, which found that CO adsorption was enhanced with a Pd content increase.

### 2.2. DFT Computational Analysis

#### 2.2.1. Adsorption of CO on the Surfaces of Pd(111), Pt(111), Rh(111), and Ag(111)

Molecule adsorption is the initial step in a heterogeneous reaction. Adsorption energy and adsorption geometry significantly influence the velocity of the reactions. Therefore, in order to obtain deep insight into the mechanism of color change during CO exposure over the catalyst, we first performed DFT calculations of CO adsorption on the Pd surface. Other noble transition metals such as Pt, Rh, and Ag were also selected for the CO adsorption calculations to compare the results on the Pd surface. The experimental studies of color changes between these four metal-loaded TiO_2_ catalysts were also conducted in the following section. The (111) surfaces were constructed for these four metals for the CO adsorption calculations because the (111) surface is recognized as the most active surface for metal-loaded catalysts. The values and directions of electron transfer between CO and the surfaces of these four catalysts were also calculated.

**Figure 5:**
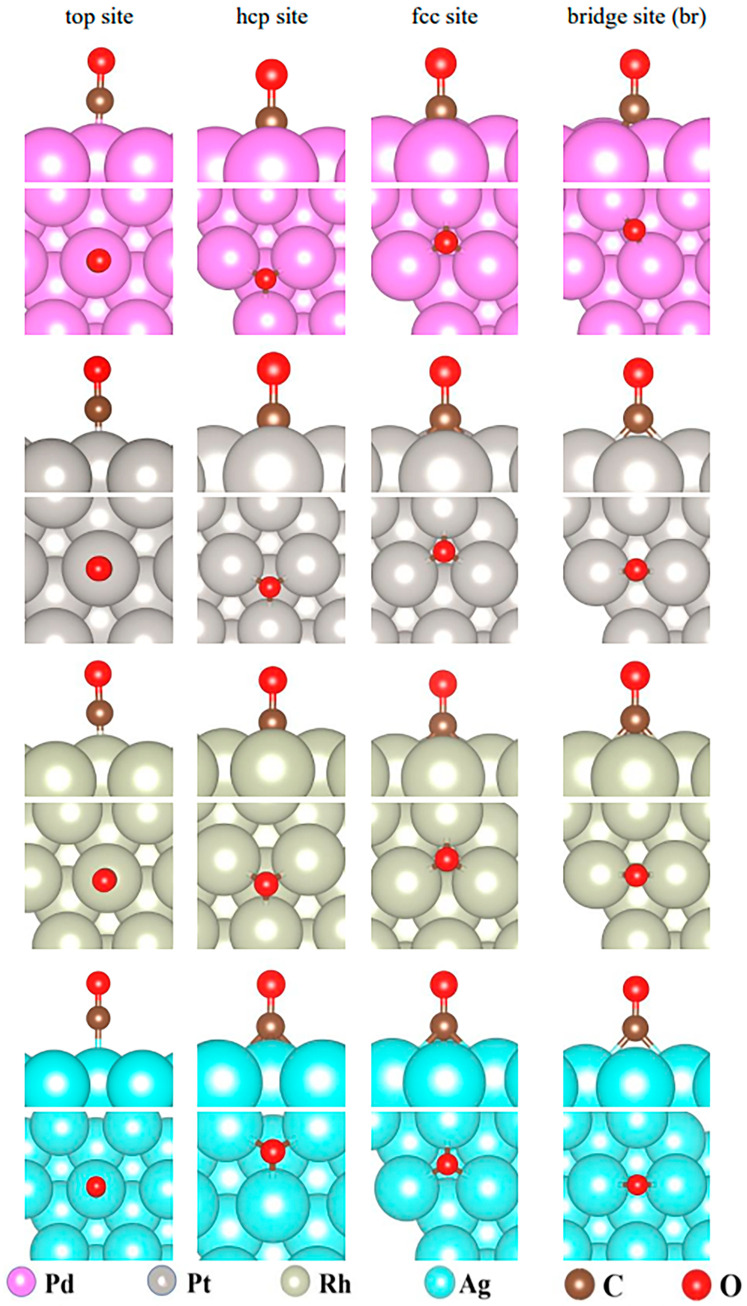
Adsorption geometries of CO on the top, hcp, fcc, and bridge sites of Pd(111), Pt(111), Rh(111), and Ag(111) surfaces. Pink, gray, light green, cyan, brown, and red spheres represent atoms of Pd, Pt, Rh, Ag, C, and O, respectively.

The most stable adsorption configurations of CO on these four surfaces are shown in Figure 5, and the corresponding adsorption energies are shown in Table 2. A negative adsorption energy means the adsorption is exothermic. The more negative the value, the more energy is released and the stronger the interaction presented. It can be clearly found from Table 2 that the CO adsorption on the Pd(111) surface at the hcp site had the largest energy of −1.99 eV, suggesting that CO had the strongest interaction with the Pd surface. The corresponding bond lengths of three Pd-C bonds were 2.07, 2.07, and 2.08 Å. The CO adsorption energy at the fcc site over Pd(111) was −1.94 eV, which was close to that at the hcp site. The largest CO adsorption energies over Pt(111), Rh(111), and Ag(111) were −1.85 eV (at hcp site), −1.81 eV (at top site), and −0.07 eV (at the top and fcc sites), respectively. All these energies were smaller than that on the Pd(111) surface. Therefore, the metal Pd presented the strongest attraction for CO compared with other noble metals Pt, Rh, and Ag. The CO adsorption energies on the Ag(111) surface were as small as −0.06 and −0.07 eV, much smaller than those on Pd(111), Rh(111), and Ag(111), suggesting that Ag had the weakest attraction for CO. The corresponding bond length of Ag-C at the top site was 2.17 Å, which was also longer than those on Pd(111). The largest CO adsorption energies on the Pt(111) and Rh(111) surfaces were 0.1 eV, smaller than that on Pd(111). Compared with Pd, the noble metals Pt and Rh may have a similar CO adsorption capacity. As a result, the interactions between CO and the noble metals were in the order of Pd > Pt > Rh >> Ag.

**Figure 6:**
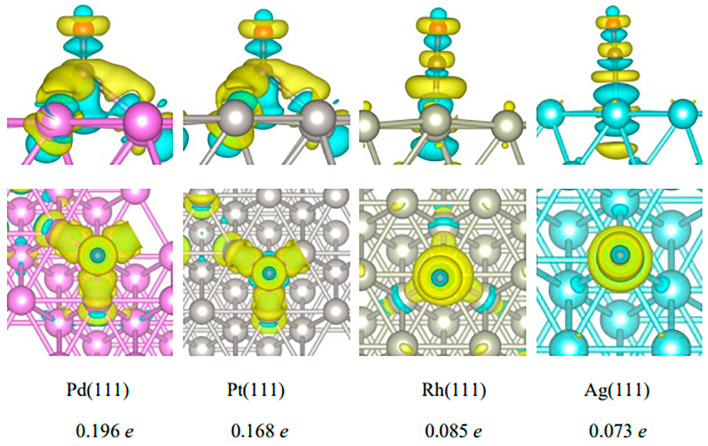
Differential charge density of CO on Pd(111), Pt(111), Rh(111), and Ag(111) surfaces. Yellow and cyan isosurfaces indicate electron accumulation and depletion, respectively. Pink and gray spheres represent Pd and Pt atoms, respectively. The value in the lower represents the electrons transferred from the metal surface to CO.

Bader charge analysis was used to calculate the differential charge density and the charge transfer of CO over the Pd(111), Pt(111), Rh(111), and Ag(111) surfaces, and the corresponding results are presented in Figure 6. A yellow isosurface represents electron enrichment, and a cyan isosurface represents charge depletion. Figure 6 shows that tiny electrons were shifted between CO and surfaces of Rh(111) and Ag(111), which had values as low as 0.085 and 0.073 *e*, respectively. CO had no obvious charge transfer on the surface of Rh(111) and Ag(111). Compared with Rh(111) and Ag(111), the electron transfers between CO and the surface of Pd(111) and Pt(111) were more intense. Electrons were enriched from the Pd(111) and Pt(111) surfaces to CO, and the charge transfer amounts were 0.196 *e* and 0.168 *e*, respectively. The molecule CO was an electron acceptor, while the metal surfaces were electron donators. The number of charges transferred from Pd(111) to CO was the largest, confirming that the interaction between CO and Pd was the strongest. This is consistent with the abovementioned results of CO adsorption energy. Compared with the Pt(111), Rh(111), and Ag(111) surfaces, both the charge transfer and the adsorption energy of CO on the surface of Pd (111) were the largest, and the interaction between CO and Pd(111) was the strongest. The greater the electron transfer from the surface of Pd (111) to CO, the easier the valence state change, resulting in an easier and faster color change after CO exposure. Therefore, it was concluded that Pd was the best noble metal active component of the supported TiO_2_ catalyst for highly sensitive gasochromic CO.

#### 2.2.2. Adsorption of CO on the Surface of PdO

**Figure 7:**
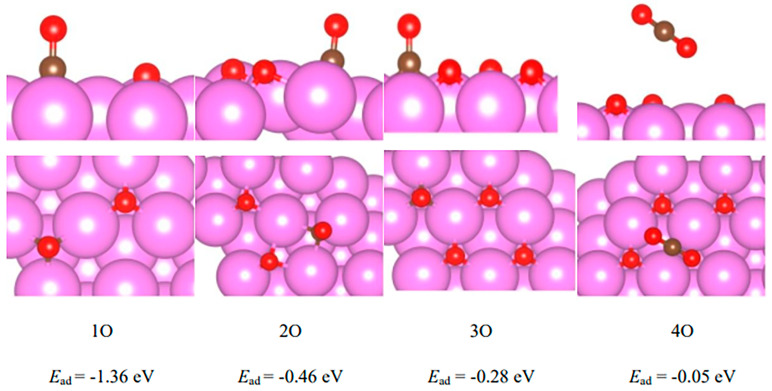
Adsorption geometries and energies of CO on PdO surface. 1O, 2O, 3O, and 4O simulate different oxidation states of the top-layer surface of PdO. Pink, brown, and red spheres represent atoms of Pd, C, and O, respectively.

The hcp site of the most stable adsorption configuration of CO on the Pd(111) surface was selected, and the adsorption energy calculation and analysis of CO on the PdO surface were further calculated. The results are shown in Figure 7. One to four O atoms were adsorbed on four Pd atoms at the top layer of Pd(111), representing different valence states of Pd. When there were 4 O at the top layer, the Pd/O ratio was 1:1, which corresponded to Pd^2+^. The adsorption energy of CO on the PdO surface decreased gradually with an increase in surface O coverage. The adsorption energies on pure Pd(111) were −1.99 eV, which then reduced to −1.36 eV at the 1O surface. When the surface was covered by 2O, the adsorption energy decreased sharply to −0.46 eV. It was further decreased to −0.28 eV at 3O. When the surface was covered by 4O, corresponding to Pd^2+^, the adsorption energy was dramatically lowered to −0.05 eV. It was interesting to find that there was no CO on the 4O surface. The stable species on the 4O surface was CO_2_. This indicated that CO would be immediately oxidated to CO_2_ on an O-abundant PdO surface. Therefore, a color change would be induced by a reduction in PdO to metallic Pd on a prepared catalyst. This was in good agreement with our abovementioned XPS result, in which the proportion of Pd^0^ increased significantly after CO exposure, while the proportion of Pd^2+^ decreased obviously.

### 2.3. Catalytic Chromic Experiments

#### 2.3.1. Effect of Noble Metals

**Figure 8:**
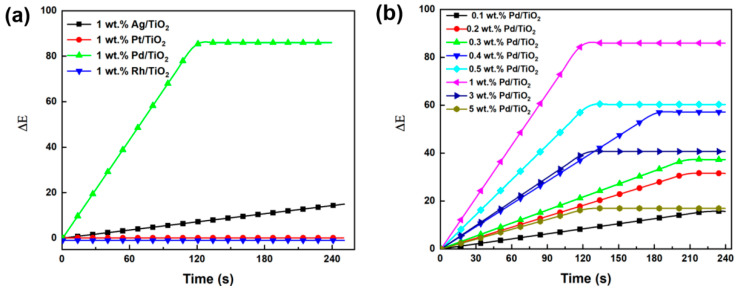
Variation in the values of color difference with respect to CO exposure time: (**a**) over Ag/TiO_2_, Pt/TiO_2_, Pd/TiO_2_, and Rh/TiO_2_ with the metal content of 1 wt.%; and (**b**) over Pd/TiO_2_ with different Pd content of 0.1–5 wt.%.

To investigate the synergistic effect between different noble metals and transition metal oxides on catalytic activity, we used the following four catalysts with different noble metal supports: 1 wt.% Pd/TiO_2_, 1 wt.% Pt/TiO_2_, 1 wt.% Rh/TiO_2_, and 1 wt.% Ag/TiO_2_. First, 0.2 g of prepared samples was loaded in a CO environment. The flow rate of CO was set to 60 mL/min. After the introduction of the gas, the chromatic aberration value ΔE, which refers to changes with time, was recorded and is shown in Figure 8a. The color change degree of each sample from high to low was Pd/TiO_2_ > Ag/TiO_2_ > Pt/TiO_2_ ≈ Rh/TiO_2_. However, no obvious color change was observed for 1 wt.% Pt/TiO_2_ and 1 wt.% Rh/TiO_2_. The highest ΔE reached to 90 over 1 wt.% Pd/TiO_2_ when the CO exposure time was kept for more than 120 s. This was consistent with the CO adsorption energy and charge transfer analysis, which also showed that CO had the strongest interaction and the largest charge transfer with Pd/TiO_2_.

#### 2.3.2. Effect of Pd Loading Content

**Figure 9:**
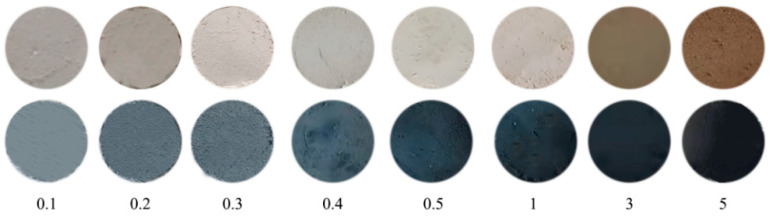
Photos of color change of Pd/TiO_2_ pre- and post-CO exposure. The values in the lower part of the image represent the Pd content.

The effects of different contents of Pd on the gas-chromic performance of materials were studied. The results are shown in Figure 8b. Samples with a Pd content of 0.1 wt.%, 0.2 wt.%, 0.3 wt.%, 0.4 wt.%, 0.5 wt.%, 1 wt.%, 3 wt.%, and 5 wt.% were selected for color change experiments in the CO environment. The result shows that the amount of ∆E is proportional to the Pd content. The chromatic aberration values reached a maximum value of 90 when the content of Pd was 1 wt.%. The color contrasts of Pd/TiO_2_ before and after the reaction with CO are shown in Figure 9. It can be clearly seen from the figure that the color difference before and after the reaction of 1 wt.% Pd/TiO_2_ is the most obvious. Therefore, 1 wt.% Pd/TiO_2_ can be used as an effective gas detection material for the detection of CO in air at room temperature.

## 3. Experimental

### 3.1. Synthesis of Pd/TiO_2_ Catalyst

Pd/TiO_2_ was prepared by the impregnation method and using palladium chloride (60% PdCl_2_, Shanghai Aladdin Biochemical Technology Co, Ltd., Shanghai, China), hydrochloric acid (1 mol∙L^−1^ HCl), and titanium dioxide (TiO_2_, AR, Shanghai Aladdin Biochemical Technology Co, Ltd.) as the precursor, pH regulator, and support, respectively.

Taking the 1 wt.% Pd/TiO_2_ catalyst as an example, 0.05 g of PdCl_2_, 2.97 g of TiO_2_, and 3 mL of HCl were added into 5 mL of deionized water and heated and stirred to obtain a homogeneous mixture. The mixture was stood at room temperature for 3 h. Then, it was stirred and baked for 30~60 min. The resulting powdered mixture was dried overnight at 120 °C and then calcined in air at 400 °C for 3 h at a heating rate of 3 °C/min. The obtained light yellow solid grain was the 1 wt.% Pd/TiO_2_ sample. For comparison, the Pt-, Rh-, and Ag-supported TiO_2_ composites were prepared using the same method. Finally, the obtained solid grain was denoted as 1 wt.% Pd/TiO_2_, 1 wt.% Rh, and 1 wt.% Ag. In addition, 0.1 wt.% Pd/TiO_2_, 0.2 wt.% Pd/TiO_2_, 0.3 wt.% Pd/TiO_2_, 0.4 wt.% Pd/TiO_2_, 0.5 wt.% Pd/TiO_2_, 1 wt.% Pd/TiO_2_, 2 wt.% Pd/TiO_2_, and 3 wt.% Pd/TiO_2_ were also prepared to study the effect of Pd loading contents on color sensitivity performance.

### 3.2. CO Gasochromic Evaluation

A Hikvision E14a camera was used to record the color change of the Pd/TiO_2_ sample after CO exposure. The color difference before and after CO exposure was calculated using the YOLOv5 algorithm [38,39,40]. Firstly, the initial pixel value with no CO exposure to Pd/TiO_2_ was recognized as E_0_. Then Pd/TiO_2_ was exposed to CO for a period. The value of all pixels in the recognition area during the color change caused by CO exposure was measured. The data were obtained by adding an initial 49 data points, and then the average value *E_i_* was calculated by Equation (1). After that, the three components of pixel *E_i_* in the BGR color space were recorded as *B_i_*, *G_i_*, and *R_i_*, and the color difference value *ΔE* was calculated by Equation (2). Equations (1) and (2) were shown as:

(1) $$E_{i}=\frac{\sum_{i-49}^{i}E_{i}}{50}$$

(2) $$∆E=\sqrt{{\left(B_{i}-B_{0}\right)}^{2}+{\left(G_{i}-G_{0}\right)}^{2}+{\left(R_{i}-R_{0}\right)}^{2}}$$

### 3.3. Catalyst Characterization

Specific surface areas of the prepared Pd/TiO_2_ catalysts were calculated using the N_2_ adsorption–desorption test. The structural characteristics of the samples were analyzed using an ASAP-2460 automatic adsorption instrument manufactured by Mike Corporation (Fukushima, Japan). The specific surface area, total pore volume, and pore size distribution of the samples were calculated using the BJH method and the DFT method, respectively. The specific surface areas of the catalysts were calculated using the Brunauer–Emmett–Teller (BET) method, while the average pore size (D_p_) and total pore volume (V_total_) were determined by the Barrett–Joyner–Halenda (BJH) method. The catalysts underwent vacuum degassing at 300 °C for 12 h firstly, followed by cooling to room temperature. Subsequently, the N_2_ adsorption and desorption tests were carried out at 77 K.

The CO adsorption capacity of catalysts can be studied by temperature-programmed desorption in CO (CO-TPD). The 25 mg catalyst was put into a programmed temperature-controlled heating furnace, and argon and helium were introduced, heated, and activated at 600 °C, respectively. A 10% CO/Ar mixture was then introduced and adsorbed at 800 °C. Finally, the desorption was carried out by linear heating according to a certain procedure, and the gas components in the desorption gas were detected until the desorption was completely desorbed, and the CO-TPD spectrum was obtained. The adsorption of CO on the surface of the catalyst CO-TPD was conducted using the TP-5080 automatic dynamic adsorption instrument (Tianjin Xianquan, Tianjin, China). The crystal structure of the sample was analyzed using Thermo Scientific’s K-Alpha X-ray photoelectron spectrometer (XPS) (Norristown, PA, USA). The XPS was measured by a monochromatic Al Kα (hv = 1486.6 eV) ray source with a power of 150 W and a beam spot of 400 μm.

The surface groups and chemical bonds of the catalysts were characterized using a Fourier infrared spectrometer (FT-IR). A total of 10 mg of the catalyst was taken and made into a transparent sheet using the tableting method, and then the infrared spectrogram was recorded using a DTGS KBr detector (Thermo Fisher Scientific, Norristown, PA, USA).

## 4. Conclusions

A Pd/TiO_2_ visible gas-chromic material for the fast room-temperature detection of CO was reported. The effect of these materials on CO was assessed by loading different kinds and different contents of noble metals (Pd, Pt, Rh, and Ag) onto TiO_2_. When the Pd content was 1 wt.%, the color change ∆E reached a maximum value of 90. DFT calculations showed that CO had the largest adsorption energy of −1.99 eV at a hcp site of Pd(111). The charge transfer between CO and the Pd(111) surface was 0.196 *e*. CO acted as an electron acceptor, while the Pd surface acted as an electron donator. Systematic catalyst characterizations confirmed that the color change was caused by a reduction in surface Pd^2+^ to Pd^0^. This work provides some inspiration for the technological development of CO fast detection at room temperature.

## Figures and Tables

**Table 1:** Textural properties of the catalysts.

Catalyst	S_BET_ (m^2^·g^−1^)	V_total_ (cm^3^·g^−1^)	D_P_ (nm)
0.5 wt.% Pd/TiO_2_	75.2	0.44	23.6
1 wt.% Pd/TiO_2_	76.8	0.45	23.3

**Table 2:** Adsorption energy (*E*_ad_) and bond length (D_M-C_) of CO on Pd(111), Pt(111), Rh(111), and Ag(111) surfaces.

Metal Surface	Adsorption Site	*E*_ad_ (eV)	D_M-C_ (Å)
Pd(111)	top	−1.43	1.85
	hcp	−1.99	2.07, 2.07, 2.08
	fcc	−1.94	2.08, 2.08, 2.08
	br	−1.83	1.99, 2.00
Pt(111)	top	−1.72	1.84
	hcp	−1.85	2.10, 2.12, 2.12
	fcc	−1.78	2.12, 2.13, 2.13
	br	−1.83	2.02, 2.02
Rh(111)	top	−1.81	1.84
	hcp	−1.79	2.09, 2.09, 2.10
	fcc	−1.68	2.10, 2.10, 2.14
	br	−1.70	2.03, 2.03
Ag(111)	top	−0.07	2.17
	hcp	−0.06	2.34, 2.32, 2.32
	fcc	−0.07	2.32, 2.31, 2.32
	br	−0.06	2.25, 2.25

## Data Availability

Data available on request due to restrictions, e.g., privacy or ethical.
